# Performance of self-diagnosis and standby treatment of malaria in international oilfield service employees in the field

**DOI:** 10.1186/1475-2875-7-128

**Published:** 2008-07-14

**Authors:** Anna H Roukens, Johannes Berg, Alex Barbey, Leo G Visser

**Affiliations:** 1 Infectious Diseases, Leiden University Medical Center (LUMC), 2300 RC, Leiden, The Netherlands; 2 Shell International B.V.-Corporate Affairs Health, 2501 AN, The Hague, The Netherlands; 3 Schlumberger Limited, Rue René Barthelemy, 92120, Montrouge, France

## Abstract

**Background:**

Falciparum malaria remains a major occupational illness that accounts for several deaths per year and numerous lost working days among the expatriate population, working or living in high-risk malarious areas. Compliance to preventive strategies is poor in travellers, especially business travellers, expatriates and long-term travellers.

**Methods:**

In this cross-sectional, web-based study the adherence to and outcome of a preventive malaria programme on knowledge, attitudes and practices, including the practice of self-diagnosis and standby treatment (curative malaria kit, CMK) was evaluated in 2,350 non-immune expatriates, who had been working in highly malaria endemic areas.

**Results:**

One-third (N = 648) of these expatriates visited a doctor for malaria symptoms and almost half (29 of 68) of all hospitalizations were due to malaria. The mandatory malaria training for non-immunes was completed by 92% of those who visited or worked in a high risk malaria country; 70% of the respondents at risk also received the CMK. The malaria awareness training and CMK significantly increased malaria knowledge [relative risk (RR) of 1.5, 95%CI 1.2–2.1], attitudes and practices, including compliance to chemoprophylaxis [RR = 2.2, 95%CI 1.6–3.2]. Hospitalization for malaria tended to be reduced by the programme [RR = 0.4, 95%CI 0.1–1.1], albeit not significantly. Respondents who did not receive instructions on the rapid diagnostic test were two times [RR = 2.3, 95%CI 1.6–3.3] more likely to have difficulties. Those who did receive instructions adhered poorly to the timing of repeating the test. Moreover, 6% (31 of 513) of those with a negative test result were diagnosed with malaria by a local doctor. 77% (N = 393) of the respondents with a negative test result did not take curative medication. 57% (252 of 441) of the respondents who took the curative medication that was included in the kit did not have a positive self-test or clinical malaria diagnosis made by a doctor.

**Conclusion:**

This survey demonstrated that a comprehensive programme targeting malaria prevention in expatriates can be effectively implemented and that it significantly increased malaria awareness.

## Background

Every year, *Plasmodium falciparum *infects 300 to 500 million persons, and kills between one and two million. Particularly sub-Saharan Africa, parts of South America and South-East Asia are affected. Falciparum malaria is also a major occupational illness that accounts for several deaths per year and numerous lost working days among the expatriate population, working or living in high-risk malarious areas. Approximately 1% of all non-immune travellers who acquire *P. falciparum *infection die [1].

Increasing awareness, personal protection measures against mosquito bites, chemoprophylaxis, and early diagnosis and treatment are the mainstay of prevention against falciparum malaria. Compliance to these preventive strategies is poor in travellers, especially business travellers, expatriates and long-term travellers [2]. Moreover, the diagnosis of malaria is often not immediately considered in returning travellers, resulting in treatment delay and subsequent higher morbidity [3].

In 2003, a preventive programme for international employees and contractors working in malaria endemic areas was set up by an oilfield service company to enhance awareness on the dangers of malaria, and to reduce its morbidity and mortality. The cornerstones of this preventive programme were a malaria awareness training programme and provision of a curative malaria kit, which contained dipstick-based strips for self-diagnosis and emergency standby medication for self-treatment of falciparum malaria. In an initial survey, this programme was rated very good to excellent by more than 60% of the respondents [4].

In this cross-sectional study by web-based questionnaire, the adherence to this preventive malaria programme, and the practice of self-diagnosis and standby treatment of presumptive falciparum malaria in the field was evaluated.

## Methods

### Malaria prevention programme

The malaria prevention programme consists of the following components:

1. Malaria training for non-immunes. This training was mandatory for all non-immune international oilfield service company employees. Any person who had left a malaria endemic country for more than six months was considered non-immune to malaria.

2. Arrival packages were assigned to employees with high-malaria-risk destinations, according to the WHO malaria country definition [5]. A quiz was designed to enhance the awareness of expatriate workers on the risks of malaria and the possible preventive measures.

3. At all malarious locations appropriate preventive measures were provided, including insecticide treated bed nets, routine malaria prophylaxis, insect repellents and insecticide treatments to kill mosquito larvae in company facilities and residences.

4. Malaria hot line. A toll-free telephone line, staffed by multilingual doctors who were specialized in tropical diseases, was available 24 hours a day, seven days a week.

5. A curative malaria kit (CMK) with hands-on training. This kit was developed to address emergency cases of suspected malaria in which an individual was more than 24 hours away from a medical centre. The kit consisted of forehead temperature strips, three dipstick-based, immunological antigen-capture self-tests for falciparum malaria (Paracheck Pf^® ^or Core Malaria Pf^®^, depending on availability), and curative medication (Coartem^®^: artemether/lumefantrine). If the self-test was positive the infected person was instructed to start taking the curative medication (four tablets every morning and four tablets every evening for three days), and seek medical assistance as soon as possible. In case of a negative test result, the blood test was to be repeated 12 hours later.

### Web-based questionnaire

To evaluate the malaria prevention programme, an e-mail invitation to answer a web-based questionnaire (NetQuestionnaires version NETQ 6.0, the Netherlands) was sent in July 2007 to 8,380 oilfield service company employees, who were registered as non-immune to malaria, and who might have travelled to, lived or worked in a malarious area in the last two years. The survey covered use of the programme in these preceding 24 months.

The web-based questionnaire was accessible from July to September 2007 by a unique link per addressed employee, and could be opened only once. During this period, several reminders were sent to the employees who had not yet accessed the questionnaire. The answers to the questionnaire were analysed anonymously. Gender, age and country of birth was the only personal information requested.

### Definitions

Malaria was reported as

1. 'Doctor's diagnosis of malaria'; diagnosed by a local doctor (not necessarily laboratory confirmed)

2. 'Laboratory confirmed malaria'; diagnosed by a local doctor and confirmed by laboratory

3. 'Presumptive malaria'; a positive self-test, or a clinically diagnosed or laboratory confirmed malaria.

The following subgroups were defined:

- to analyse the effect of the malaria prevention programme on several aspects concerning knowledge, attitude and practices (KAP) of malaria:

1. 'Malaria Prevention Programme' as receiving the training for non-immunes with or without CMK

2. 'No Malaria Prevention Programme' as receiving neither training nor CMK.

- to analyse the effect of the CMK on malaria KAP:

1. 'CMK' as receiving the training and the kit

2. 'No CMK' as receiving the training without the kit.

### Statistical analysis

Continuous data were analyzed with Students t-test, categorical data with Chi-square test or Fisher's exact test where appropriate. Corrected relative risk (RR) was calculated from the corrected odds ratio (OR) obtained by logistic regression. Corrected OR was recalculated into RR according to the following formula: RR = OR/((1-P)+(P*OR)), provided by Zhang and Yu [6], as the OR overestimates the RR when prevalence (P) exceeds 10%. Possible confounders for which was corrected by logistic regression are specified for all reported results. *P *values were provided for categorical data with more than two categories. Statistical analysis was performed using a computer-assisted software package (SPSS version 12.0, SPSS Inc., Chicago, IL).

## Results

The web-based questionnaire was opened by 3,575 employees, giving a total response rate of 43%. Of these respondents, 2,552 reported to have travelled to malaria endemic countries in the past 24 months, of whom 2350 (92%) completed the questionnaire entirely. Analysis of the answers of all the respondents at risk and of those at risk who completed the questionnaire did not yield different results. Therefore, only the results of the completed questionnaires are reported. The mean time to complete the questionnaire was 12 minutes and 22 seconds.

### Study population

The demographic characteristics of the studied population are listed in Table 1.

**Table 1:** Demographic characteristics of study population.

**Demographic characteristics**		N (N total 2350)	%
Gender	Male	2065	88
	Female	285	12
Age (yrs)	mean (range)	36 (19–63)	-
Continent of birth	African	733	31
	European	631	27
	South American	328	14
	Asian	301	13
	North American	174	8
	Arabic	102	4
	Oceanian	64	3
Country of birth	Malaria endemic^$^	1392	60
	Malaria non endemic	941	40
Working conditions	Outdoor*	1278	54
	Indoor	1072	46
Work status	Long term (>6 months)	1122	48
	Rotator	795	34
	Visitor	342	15
	Other (e.g. spouse)	91	4

Percentages may not add up to exactly one hundred due to rounding off. ^$^ Malaria endemic country according to the WHO (5). *Outdoor working conditions include working on a land rig or with seismic crew, off shore, on another field location or on a marine vessel.

The malaria countries visited are amongst those with the highest incidence of *P. falciparum *[6]; in descending order of frequency, the most visited countries were: Angola, Cameroon, Nigeria, India, Gabon, Sudan, Equatorial Guinea, Democratic Republic of Congo and Chad. Most respondents visited more than one endemic country; the median of endemic countries visited per respondent was 2 (range 1–105).

### Risk of malaria

A comparison was made between the cumulative incidences (CI) of malaria according to work status (Table 2). The CI of acquiring malaria increased according to work status and thus according to time spent in malaria endemic countries. In addition, chemoprophylaxis use by long term travellers was significantly lower (29%) compared to that of rotators and visitors (both 62%) (p < 0.001). In contrast to the increasing CI of malaria with a longer duration of stay, the CI of being hospitalized for malaria was similar in all groups.

**Table 2:** Cumulative incidence of malaria per 100 persons according to work status in 24 months.

		**Cumulative incidence (%) ** **of malaria in 24 months** [95%CI]			
			
		**Visitor** (N = 342)	**Rotator ** (N = 795)	**Long term ** (N = 1122)	p-value*
Malaria	Presumptive	2.3 [0.7–3.9]	6.2 [4.5–7.9]	13.7 [11.7–15.7]	<0.001
	Doctor's diagnosis	2.0 [0.5–3.5]	5.7 [4.1–7.3]	12.8 [10.8–14.8]	<0.001
	Laboratory confirmed	1.8 [0.4–3.2]	4.3 [2.9–5.7]	9.7 [8.0–11.4]	<0.001
Hospitalization for malaria	Doctor's diagnosis	0.6 [0.0–1.4]	1.6 [0.7–2.5]	1.5 [0.8–2.2]	0.6
	Laboratory confirmed	0.6 [0.0–1.4]	1.6 [0.7–2.5]	1.2 [0.6–1.8]	0.5

*p-value for malaria diagnosis was obtained by χ^2^-test and for hospitalization with Fisher's exact test. Those who responded to belong to the 'other' group (N = 91), instead of the solicited groups, were excluded as their global time of possible exposure to malaria was unclear.

Ninety percent of the respondents who reported to have had laboratory confirmed malaria acquired the disease in sub-Saharan Africa. Malaria was acquired in descending order of frequency in Sudan, Nigeria, Equatorial Guinea, Angola, Chad, Cameroon, Republic of Congo, Gabon, Ivory Coast, India, Benin, Somalia, Uganda and Peru. The considerable burden of malaria in this population was demonstrated by the fact that one-third (N = 648) of all respondents visited a doctor for malaria symptoms and almost half (29 of 68) of all hospitalizations were due to laboratory confirmed malaria.

### Malaria prevention programme

**Figure 1:**
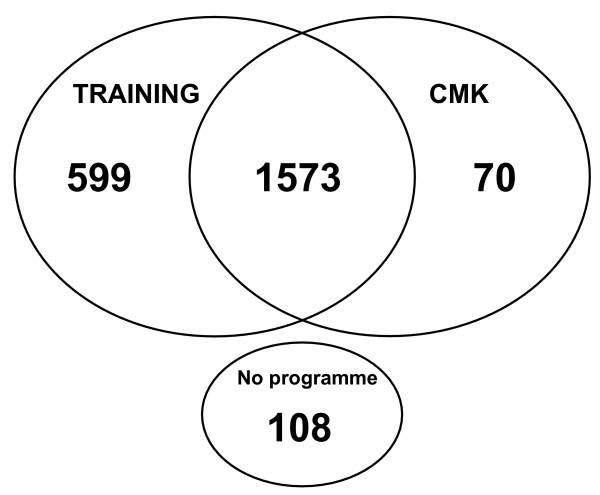
**Distribution of the Malaria Prevention Programme in population at risk (N = 2350).** Numbers represent number of respondents receiving this part of the programme. Training = Training for non-immunes, CMK = Curative Malaria Kit.

The mandatory malaria training for non-immunes was completed by 92% of those who visited or worked in a high risk malaria country. Overall, 70% of the respondents at risk also received the CMK (Figure 1). Seventy-five percent (N = 1229) of respondents who received the CMK were instructed in how to use it, and all (98%) considered the instructions to be clear.

Multivariate analysis showed that respondents who were born in a malaria endemic country were two times less likely to receive the malaria prevention programme [RR 2.0, 95% CI 1.4–2.7]. In addition, women were less likely to receive the CMK [RR 1.4, 95% CI 1.1–1.7]. The effect of the malaria prevention programme and the CMK on malaria KAP was therefore corrected for these variables. The distribution of the programme was not influenced by work status, i.e. whether employees were long-term workers, rotators or visitors, neither by working indoors or outdoors.

Respondents receiving the malaria prevention programme reported a twofold higher use of malaria chemoprophylaxis (47% vs. 19%) and had significantly more knowledge about malaria. A similar effect was observed for those who only received the CMK. Those who did not receive the programme were twice as likely not to consider malaria as a threat, nor to take additional anti-mosquito measures (Table 3).

**Table 3:** Effect of Malaria Prevention Programme on malaria KAP

	**Malaria Prevention Programme**				**Curative Malaria Kit**			
						
Malaria Knowlegde, Attitudes and Practices	Training and/or CMK	No training and no CMK	RR [95%CI]	Corrected RR^# ^ [95%CI]	Training with CMK	Training w/o CMK	RR [95%CI]	Corrected RR^#^ [95%CI]
Chemical prophylaxis use n/N (%)	1049/2242 (47)	21/108 (19)	2.4 [1.8–3.0]	2.5^$^ [1.9–3.2]	861/1573 (55)	171/599 (29)	1.9 [1.7–2.1]	1.9^$ ^ [1.6–2.0]
Not considering malaria as a threat n/N (%)	126/1873 (7)	13/97 (13)	0.5 [0.3–0.9]	0.4 [0.2–0.8]	69/1337 (5)	53/480 (11)	0.5 [0.3–0.7]	0.5 [0.3–0.7]
Not inclined to take anti mosquito measures n/N (%)	103/1746 (6)	16/78 (21)	0.3 [0.2–0.5]	0.3 [0.2–0.5]	58/1236 (5)	39/453 (9)	0.6 [0.4–0.8]	0.5 [0.3–0.8]
Correct malaria knowlegde* n/N (%)	977/2242 (40)	28/108 (26)	1.7 [1.3–2.1]	1.6 [1.2–2.1]	745/1573 (47)	202/599 (34)	1.4 [1.3–1.6]	1.4 [1.2–1.5]

RR = Relative Risk.

^#^Corrected for malaria endemic country of birth.

^$^Additionally corrected for work status (long term traveller, rotator, etc.).

*Knowlegde was examined by multiple choice question on the maximum incubation time of *Plasmodium falciparum*. The denominators for questions on considering malaria as a threat and inclination to take anti-mosquito measures vary, as answers reporting 'neutral' were not used for analysis.

Despite the increased use of chemoprophylaxis by the total group receiving the CMK, a small group (14%, N = 226) thought that having the CMK made regular malaria chemoprophylaxis unnecessary. The use of chemoprophylaxis in this group was 49% in comparison to 60% of those who felt prophylaxis remained necessary with CMK use (p = 0.001).

### Use of self-test

One-third (N = 575) of the respondents who had received the CMK performed the malaria self-test contained in the CMK for presumptive malaria. Forty-nine test results were positive (defined as a positive test result at first or repeated testing), 508 negative and 18 invalid. Two-thirds (N = 378) repeated the test, giving a similar result in 79% (19 of 24), 99% (338 of 344) and 40% (4 of 10), respectively. Although it was instructed to repeat the test after 12 hours if the result was negative, only 55% (N = 189) adhered to this instruction.

Fifteen percent of the respondents reported having difficulties in using the self-test. Among those, the most frequently reported difficulties were pricking the finger and placing the blood drop on the test strip (Table 4).

**Table 4:** Difficulties with self-test contained in the CMK reported by respondents who used the test.

Difficulties with self-test		N (%)
Respondents reporting difficulties N performing self-test = 575		85 (15)
		
Difficulties N total = 85	Finger prick	50 (59)
	Placing blood drop	24 (28)
	Result interpretation	15 (18)
	Identifying lines	13 (15)
	Technical problem kit	12 (14)
	Instructions	10 (13)
	Adherence to waiting time	2 (2)
	Too ill to perform test	1 (1)

More than one difficulty could be reported per respondent.

Respondents who did not receive instructions with the self-test were two-times more likely to have difficulties [RR = 2.3, 95%CI 1.6–3.3] and three-times more likely to have an invalid test [RR = 2.9, 95%CI 1.0–8.5]. Respondents with difficulties were 30 times more likely to have an invalid test result [RR = 29.6, 95%CI 8.2–106.4], after correction for possible confounding of receiving instructions.

### Use of medical care

Almost twice as many respondents with a positive result visited a doctor for malaria symptoms, and a positive test indicated a tenfold higher risk of being diagnosed with malaria. On the other hand, 6% (31 of 513) of those with a negative test result were still diagnosed with malaria by a local doctor, although this diagnosis was 1.5 times less likely to be confirmed by a laboratory (Table 5).

**Table 5:** Influence of test performance and result (positive if first or repeated test result was positive) on doctor visit and malaria diagnosis and hospitalization.

	CMK received				
	
	Self-test performed				Self-test not performed N = 1068
		
	Self-test result		RR [95%CI]	Corrected RR^# ^ [95%CI]	
				
	Positive N = 49	Negative N = 508			
Visited doctor for malaria symptoms N Yes (%)	40 (82)	233 (46)	1.8 [1.5–2.0]	1.8 [1.4–2.0]	177 (17)
Doctor's diagnosis malaria N Yes (%)	33 (67)	31 (6)	11.0 [8.3–13.2]	10.3 [7.4–12.8]	59 (6)
Laboratory confirmed malaria N Yes (%)	28* (85)	18* (58)	1.5 [1.1–1.7]	1.5 [1.2–1.7]	47* (80)
Hospitalization for malaria N Yes (%)	4* (12)	4* (13)	0.9 [0.2–2.9]	1.0 [0.2–3.0]	8* (14)

RR = Relative Risk.

^#^RR is corrected for malaria endemic country of birth.

*Denominator is respondents with a doctor's diagnosis of malaria.

When hospitalization was employed as an indicator for severity of malaria, performing a test before visiting a doctor for malaria symptoms did not result in more severe malaria in comparison to immediately visiting a doctor (respectively 12%, and 14% hospitalization in those with doctor's diagnosis of malaria, p = 1.0) (table 5). In addition, respondents in whom malaria was diagnosed despite a negative test result, had a similar hospitalization rate (13%).

### Standby emergency treatment

One fifth (N = 441) of the respondents took curative medication for malaria. The origin of the curative medication was mostly the CMK (39%) or a local hospital (35%). Ninety percent (N = 44) of the respondent with a positive test result and 22% (N = 115) of the respondents with a negative test result took curative medication.

Fifty seven percent (N = 252) of respondents who took curative medication did not have presumptive malaria. The source of this inappropriately used curative medication was two times more likely to be the CMK than the medication used by those with presumptive malaria (50% vs. 25% respectively).

### Effect of the malaria prevention programme on the outcome of malaria

1.1% (N = 25) of the respondents who had received the malaria prevention programme was hospitalized for laboratory confirmed malaria in comparison to 3.7% (N = 4) of those who did not receive the programme [RR = 0.3, 95%CI 0.1–0.9]. However, when corrected for birth in a malaria endemic country the risk of hospitalization was not significantly reduced [RR = 0.4, 95%CI 0.1–1.1]. There was no significant reduction in hospitalization for those who had received the CMK without training.

## Discussion

Falciparum malaria is a severe disease and international employees and contractors working in highly endemic malarious areas are particularly at risk. In this study, it was found that one per 200 employees per year was hospitalized because of laboratory confirmed malaria, and 90% of malaria was acquired in sub-Saharan Africa. The self-test was positive in 8% of the respondents. Malaria was also diagnosed by a medical doctor in 6% of the repondents with a negative test. The malaria awareness training and self-diagnosis and treatment had a significant positive effect on knowledge and attitude towards malaria prevention and doubled the use of malaria chemoprophylaxis. This study also suggests a reduction in hospitalization for malaria, thus reducing malaria associated morbidity.

Several limitations of this study require attention. First, not all employees responded to the invitation (response rate was 43%), possibly inducing a responder bias. This may have led to an overestimation of the uptake of the programme. On the other hand, some of the respondents did not or partly receive the programme, which allowed to draw seperate conclusions on the contribution of awareness training and CMK. Secondly, neither the result of the self-test nor the diagnosis of malaria by doctor or laboratory was confirmed by an independent test. Therefore, the accuracy of the interpretation of the self-test result by these febrile expatriates remains unknown. However, the endpoint of malaria was considered to be equally (in)accurate in all respondents, meaning that no diagnosis bias was introduced.

This survey showed that sub-Saharan Africa continued to pose the highest risk for the acquisition of malaria, and that long term residents are at the highest risk to contract malaria, although they were not more likely to be hospitalized than rotators or visitors. This could reflect the experience long term travellers have with malaria, being more aware of its symptoms.

The present study confirmed that the compliance of expatriate workers to malaria prophylaxis was poor [7] and decreased with duration of stay [8]. Fifty-five percent of the respondents did not take malaria chemoprophylaxis; for comparison in travellers on vacation in high-risk areas this was 16% [2]. The availability of self-testing and standby treatment with CMK may offer non-compliant employees an additional safe guard against the serious consequences of falciparum malaria if proper medical care is not available. In addition, the introduction of the malaria awareness training and CMK significantly increased compliance to malaria prophylaxis. Despite this increased compliance, 14% of those who received the CMK thought that having the kit made regular chemoprophylaxis unnecessary. Although many of the respondents who felt this way actually did use prophylaxis. The importance of continuing prophylaxis use despite the availability of standby treatment warrants special emphasis in any educational programme.

In experienced hands, the immunological antigen-capture self-test for *P. falciparum *histidine-rich protein-2 or lactate dehydrogenase has shown to be accurate and reliable diagnostic tests for *P. falciparum *infection [9]. However, the correct performance of these dipstick-based rapid tests in febrile travelers may vary from 69% to 91% depending on whether prior instructions were given [10-12]. In the present study, 15% reported difficulties with performing the self-test, and the fact that not receiving CMK instructions was significantly associated with difficulties and invalid test results clearly underscores the need for proper instructions. Only 67% adhered to the instruction to repeat the self-test in case of a negative test result, and 55% adhered to the instructed time interval. The reason for non-adherence to these instructions is unknown. One possibility is that the self-test was not repeated because malaria symptoms had spontaneously resolved. It should be emphasized during the training that repeating the self-test within six hours after a first negative test result is unlikely to be useful as parasitaemia may still be too low to detect.

The introduction of a self-test for malaria aims at decreasing treatment delay in case of a positive test result in the absence of medical care, and at reducing the empirical use of standby treatment medication in case of fever and a negative test result. On the contrary, introduction of a self-test for malaria may increase patients' delay and lead to more severe malaria in case of false negative test result. However, the hospitalization rate of respondents with a negative test result who were subsequently diagnosed with malaria by a doctor was not significantly increased. This suggests that these patients did not have severe malaria more frequently.

Six percent of respondents who tested negative were still diagnosed with malaria. However, this diagnosis was less likely to be confirmed by a laboratory. It may reflect the possibility of overdiagnosis of malaria by a doctor, since there is anecdotal evidence that in Africa it is common practice to assume malaria, often irrespective of actual complaints [13]. The use of molecular diagnostics has the potential to overcome these limitations. When a finger prick for self-testing is performed we would recommend storing a few drops of blood on filter paper as well for PCR analysis for *P. falciparum *after returning home. This would enable future determination of true positive and true negative rates for self-testing and clinical diagnosis of falciparum malaria abroad.

The use of a self-test had a clear effect on restrictive use of standby medication: 77% of the respondents who had a negative test result did not take standby medication. Standby treatment was used not only by respondents with a positive self-test or medical diagnosis of malaria, but also in 57% who did not have a diagnosis of malaria, a number which has also been reported by others [14,15]. The CMK may have facilitated this inappropriate use, as the curative medication used by respondents without presumptive malaria originated in 50% from the CMK. This aspect will require future scrutiny; the improper use of self-treatment may result in unnecessary exposure to side-effects and in a delay of diagnosis and treatment of other potentially life threatening diseases.

## Conclusion

This survey demonstrates that, with proper instruction and training, a preventive malaria programme can contribute to the awareness of the risks of this disease. The components of this programme that deserve attention are the instructions on the performance of the self-test, the correct use of the curative medication and the need to seek medical care regardless of use of CMK. As it is impossible to make all travellers, irrespective of their purpose or duration of travel, adhere one hundred percent to every preventive measure, the contribution of the separate components which raise awareness and protection is cumulative. For those travellers considered to be exposed to higher risks of infection, such as expatriates, this malaria prevention programme certainly is such a component. Its strength lies in the multi-step design, in which a missed step is pre-empted by the next.

## Authors' contributions

AR designed the questionnaire, acquired and analysed the data and wrote the manuscript. JB designed the questionnaire, discussed the data and the manuscript. AB designed the questionnaire, acquired the data and discussed the manuscript. LV designed the questionnaire, acquired and analysed the data and wrote the manuscript.
